# “We Don't Normally Go Down This Avenue; This Is Normally Taboo”: Using Co‐Design to Develop a Training Intervention for Spiritual Health in Primary Care

**DOI:** 10.1111/hex.70737

**Published:** 2026-06-21

**Authors:** Ishbel Orla Whitehead, Mark Adley, Alexandra Thompson, Elizabeth Westhead, Marina Politis, Philip Mordue, Amy O'Donnell, Barbara Hanratty

**Affiliations:** ^1^ Population Health Sciences Institute, Campus for Ageing and Vitality Newcastle University, Newcastle upon Tyne United Kingdom

**Keywords:** co‐design, co‐production, person‐based approach, primary care, social prescribing, spiritual health, spirituality

## Abstract

**Introduction:**

Spiritual health is an important component of holistic health and social care provision; however, previous research highlights a training gap in this area. The SHARP (Spiritual Health Awareness and Recommendations in Primary Care) project used co‐design processes informed by the Person‐Based Approach (PBA) to develop a training intervention to address this gap. This paper evaluates the process of using co‐design within this sensitive, stigmatised topic area that faces challenges in terms of language, identity, power and strongly held values‐based opinions.

**Methods:**

Five co‐design workshops were held with a diverse mix of participants including GPs, social prescribers, primary care staff, chaplains, carers, patients and members of the public. Data sources included workshop transcripts, observer notes and post‐workshop participant surveys. Thematic analysis was conducted deductively in line with a co‐design evaluation framework, where we considered people‐level outcomes within and without the co‐design group, process outcomes and system‐level and sustainment outcomes.

**Results and Analysis:**

Thirty‐eight participants took part in the workshops. Analysis identified co‐design outcomes at people, group, research process and system‐level. Participants valued having space to express views on a sensitive and often taboo topic, with ‘being heard’ functioning as a prerequisite for engagement and reported high levels of engagement. Professional hierarchies and outsider status persisted despite conscious facilitation efforts, while pragmatic design choices shaped participation, continuity and collective action. The professional mix of participants supported whole‐team thinking about implementation, although recruitment of motivated participants may have limited the identification of additional barriers to change.

**Conclusion:**

The SHARP project provides important lessons on the use of co‐design in sensitive and value‐laden research topics. Specifically, for researchers to be attentive to participants' need to be heard, active management of power and hierarchy, and explicit negotiation of pragmatic constraints. Mixed‐group co‐design can support whole‐team thinking about implementation, while reflexive awareness of who is included remains critical to understanding what barriers may be surfaced or missed.

**Patient and Public Contribution:**

Patients, carers and members of the public were actively involved in the design, conduct and interpretation of this study. Public contributors and people with lived experience of primary care were recruited as equal participants within the co‐design workshops alongside clinicians and other stakeholders, where they contributed to discussions, activities and decision‐making that shaped the content, format and implementation considerations of the SHARP training intervention. Patient and public contributors also informed interpretation of findings through their reflections on workshop processes and perceived relevance to patient care. In addition, members of an established patient and public involvement group were consulted prior to and following the co‐design process to advise on acceptability, burden on primary care services and communication of the intervention to patients and the public. Their feedback directly informed refinement of the intervention and dissemination materials.

**Abbreviations**
CFBOs: Community Faith Based Organisations
GPs: general practitioners
NHS: National Health Service
SHARP: Spiritual Health Awareness and Recommendations in Primary care

**Table:** 

What is ‘spiritual health’? Spiritual health is a broad concept, as diverse as people themselves. As part of this survey, spiritual health was not defined and left to the participant to define themselves. The authors use a definition of spiritual health developed by UK General Practitioners and further developed with social prescribers: self‐actualisation, peace, purpose and meaning; transcendence, connectivity and relationships beyond the self; and expressions of spirituality.

## 1 Introduction

Spiritual health is an integral aspect of holistic well‐being and has been associated with positive health outcomes [1, 2, 3, 4]. When spiritual health needs are neglected, there can be detrimental effects including increased symptom burden and distress [5, 6, 7, 8]. Primary care has an explicit focus on providing holistic, or whole person care to patients, and aims to address health within its biopsychosocial‐spiritual contexts [9]. However, those working in primary care often express a lack of confidence and preparedness when it comes to discussing spiritual health with patients [10, 11, 12, 13, 14]. This is compounded by a sense of pluralistic ignorance, where clinicians may recognise potential benefits of addressing spiritual health but may feel uncertain about how to approach the topic within patient consultations due to the perceived stigma and/or professional ambiguity surrounding spiritual health [13, 14]. Spiritual health discussions, while having real‐world meaning for staff [15, 16], appear to feel difficult, polarised, stigmatised or taboo [12, 13, 14, 15, 16, 17], and there is a lack of training in place to support these [10, 11, 12, 13]. Patients may be waiting for clinicians to raise the topic [18], which could cause inequity of access to holistic care [19]. The evidence base for spiritual assessment in primary care, including patient desire for these conversations and the use of structured tools such as the HOPE tool, is explored in detail in our previous work [10, 12, 13, 14, 16, 20].

Co‐design in health and social care has been used to support the creation of interventions that both address real‐world clinical problems and are also feasible and acceptable to implement [21, 22]. The Person‐Based Approach (PBA) to co‐design has been used to guide professional training in stigmatised or contested topics [21, 22]. The PBA offers a framework to guide the co‐design process that focuses on understanding and integrating the lived experiences of stakeholders who will use or benefit from the intervention or innovation (in this situation, training), including care professionals, patients and carers. The three strands of the existing evidence base, relevant theory and the in‐depth appreciation of user experience are plaited together to iteratively and systematically create a practical, acceptable intervention, integrating the evidence and theory. Within the SHARP project, we used the evidence base [10, 11, 12, 13, 14, 23, 24, 25, 26, 27] with Normalisation Process Theory (NPT) [28], to understand how the lessons from training on spiritual health could be embedded and sustained within NHS primary care [29]. Both NPT and PBA have been used to support the development of interventions within other stigmatised topics, such as HIV research [30, 31], people who inject non‐prescribed drugs [32, 33], and gender‐based care [34]. Within the SHARP project, we have used co‐design, specifically the PBA, with NPT, to create the SHARP training that aims to change how staff in primary care approach spiritual health.

However, although co‐design is increasingly used in health intervention development, there is little evidence about its application to spiritual health, which can be perceived as polarised, stigmatised or taboo. This paper aims to address this gap in the methodological literature and to add to existing work on co‐design in stigmatised, value‐laden, high‐opinion topic areas. Specifically, this paper evaluates the co‐design process within the sensitive context of spiritual health and reflects on the challenges and strengths we encountered. Our intention is to offer insights that are relevant not only to this field but also to researchers using co‐design in other sensitive areas.

## 2 Methods

### 2.1 Design and Setting

The PBA was used to co‐design a training intervention on spiritual health in primary care, drawing on NPT [28, 33] to ensure the intervention would be coherent, implementable and aligned with real‐world clinical practice. The PBA provided a structured, yet flexible framework for establishing boundaries and collaboration [35, 36]. Psychological safety within the group was supported by ground rules and guiding principles discussed and agreed at the start of each workshop (Appendix 2), alongside an individual agenda (Appendix 1), and an evidence‐based working definition of spiritual health [37].

Five iterative workshops were held, two online and three in‐person at Newcastle University, United Kingdom (see Table 1) over lunchtime between April and September 2025. Each workshop focussed on a different aspect of the training and combined whole group discussions with structured small group activities, in line with PBA methods (these included user journeys where workflows, team dynamics, patient needs and organisational structures were discussed; think‐aloud exercises where personas were used to make sense of the cases and user needs, and allow concrete changes; and longer persona‐based discussions to refine the training). Adjustments to the prototype were made between workshops (see Appendix 1 for workshop agendas and Appendix 7 for Table of changes).

**Table 1:** Workshop details.

	Setting	Workshop focus	Attendees	Attendees: By role description							Survey responses	Number of repeat attendees
				General practitioner	Primary care clinician	Social prescribing worker	Spiritual health worker	Community Faith Based Organisation staff member	Member of the public or NHS patient	Any other interested person		
Workshop 1	In person	User journey exercise	9	2		2	1	1	1	2	8	N/A
Workshop 2	Online	Patient stories ‘think aloud’	5		1		2	1	1		3	1
Workshop 3	In person	Review of prototype (format)	8	2	1	1	1	1	1	1	6	3
Workshop 4	Online	Review of prototype (content)	7	2			2	2	1		3	2
Workshop 5	In person	Re‐presentation of the HOPE tool (how this should be framed and presented in the training)	9	4			1		2	2	4	5
Total			38	10	2	3	7	5	6	5	24	

### 2.2 Recruitment and Sampling

Using purposive sampling, we recruited participants with an interest in spiritual health in primary care, including general practitioners, social prescribers, primary care staff, chaplains, community faith‐based organisation (FBO) members, patients, carers and public contributors. Invitations were circulated nationally through primary care networks, professional organisations, faith‐based groups and Voice‐global (https://voice-global.org/ an organisation of patients and the public to offer lived experience input to research), from 22nd February 2025 to 22nd March 2025 (see Appendix 3 for recruitment flyer).

Eligibility criteria are shown in Table 2 below. We selected participants who expressed curiosity, openness or practical interest rather than strong opposition to the premise of spiritual health in primary care. This approach aimed to support psychological safety and avoid repeatedly re‐opening ideological debates, for example, whether spiritual health exists. Participants received a shopping voucher as a token of thanks for attending the workshop. This was common to all participants, to reinforce equality of contribution to the co‐design process, regardless of their role outside the project. Measures to identify fraudulent participants and full recruitment procedures are summarised in Appendix 4. Religious or spiritual affiliation was not collected as a formal demographic variable. Sampling prioritised diversity of role and stakeholder perspective rather than religious identity; some participants voluntarily disclosed their spiritual or religious background during workshops or evaluation forms, and this is noted where relevant in the results.

**Table 2:** Eligibility criteria.

Inclusion criteria	Exclusion criteria
People with an interest in the topic of spiritual health in primary care	People who did not disclose an interest in spiritual health.
(For in‐person workshops) able to travel to Newcastle‐upon‐Tyne	Individuals who were assessed as unable, or not willing, to understand or follow the workshop ground rules and guiding principles.
	Those who were assessed as high risk of fraudulent participation, e.g., to be outside the United Kingdom, and not have a genuine interest in spiritual health in primary care in the United Kingdom.

### 2.3 Data Sources and Collection

Three sources of data were collected via workshops: audio transcripts of workshop discussions; observer‐structured notes (Appendix 5); and post‐workshop evaluation surveys (Appendix 6). Transcription was either professional or by secure software [38]. Each workshop was attended by researchers whose role was primarily to observe group discussions and group dynamics. Survey questions asked for participant views on the relevance of the topic to their experience, whether they felt valued, and whether the workshop was interesting or the right length, with Agree, Disagree and Not Sure response options. Open‐text responses were also collected within questions such as *How did you feel about today's workshop?* The survey was developed by the research team drawing on standard co‐design evaluation practice and reviewed iteratively across the team. As a pragmatic process evaluation tool rather than a psychometric instrument, formal inter‐rater reliability testing was not conducted; this is a limitation we acknowledge.

### 2.4 Data Synthesis

**Figure 1:**
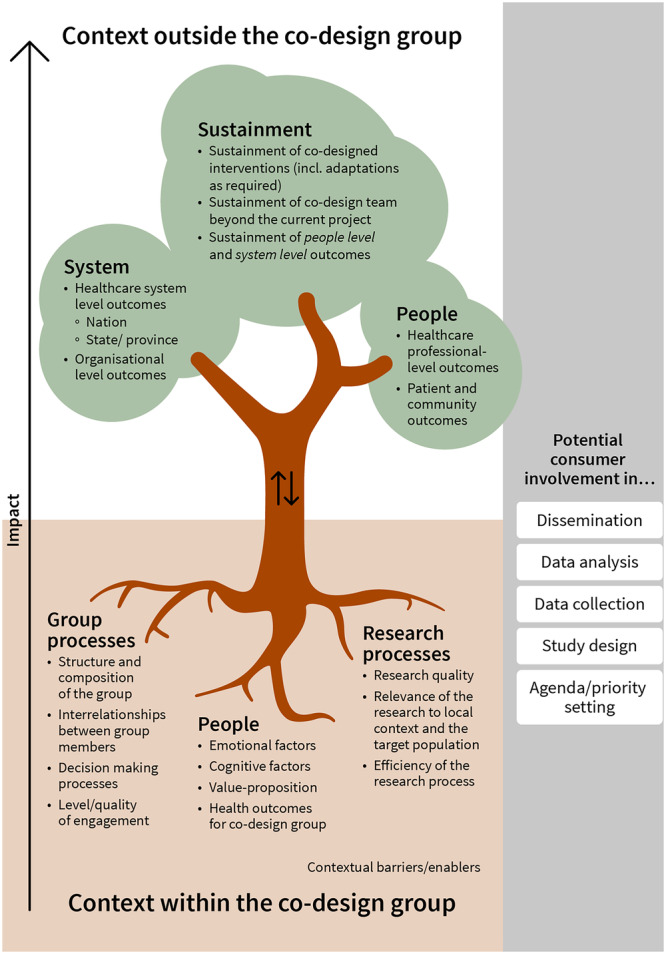
The Co‐design Evaluation Framework from Peters et al. [39] used as the deductive analytic structure for this study. The framework organises co‐design outcomes at people, group, process and system levels, and guided the thematic analysis of workshop transcripts, observer notes and evaluation surveys.

The study used a deductive thematic analysis based on the Co‐design Evaluation Framework [39] (see Figure 1) to explore how the co‐design process was used within the topic of spiritual primary care. Multiple data sources (described above) were used to triangulate the findings [40]. We analysed people‐level outcomes within the co‐design group, process outcomes and anticipated people‐ and system‐level outcomes beyond the group.

Familiarisation with the data began with attendance at the workshops, followed by individual scrutiny of the transcripts, observer notes and survey responses, with further discussion at team meetings. Coding was conducted using an AI‐enabled software system: MAXQDA software [41], and shared and reviewed by the team. Survey data were imported into Microsoft Excel [42] for analysis. Initial discussion of findings within the team prompted further deductive analysis within the Co‐design Evaluation Framework [39].

### 2.5 Underpinning Evidence

The format and content of the co‐design workshops built on our previous work within the topic of spiritual health within primary care: A survey of UK general practitioners (GPs) [37], a realist review to understand what has been tried in primary care already to improve patients' spiritual health [20], and a mixed‐methods online survey and semi‐structures interviews to obtain comprehensive data on spiritual health and its current inclusion in social prescribing [12, 13]. We used an identified definition of spiritual health in our Ground Rules (Appendix 2).

### 2.6 Underpinning Theory

**Figure 2:**
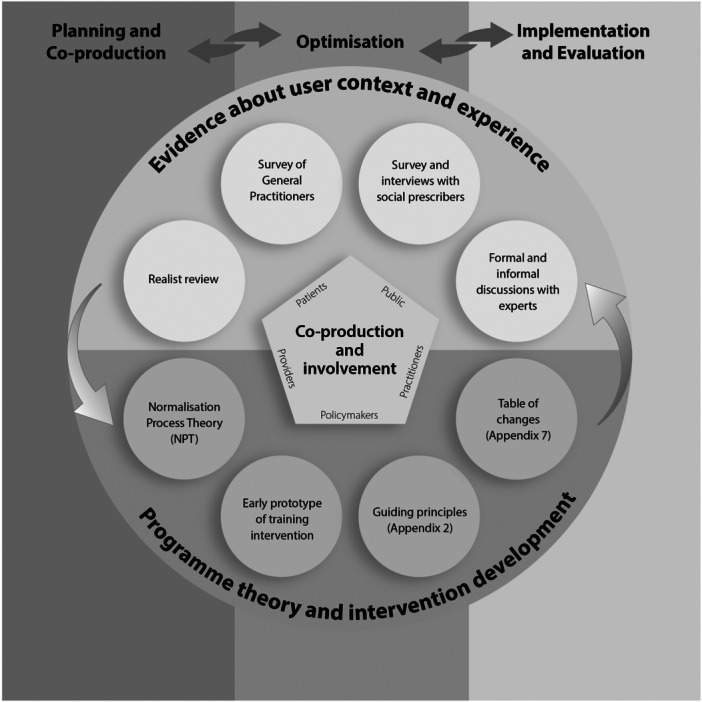
The co‐production processes used in the current study, adapted from The Person‐Based Approach [44].

We used NPT [28] to inform the development of spiritual health training that could be integrated and sustained within routine primary care practice. NPT recognises healthcare as a collective, relational activity, characterised by non‐linear and context‐dependent interactions between individuals and organisations, a perspective well suited to the contested and interpretive nature of spiritual health. This is particularly pertinent for spiritual health where relational aspects of human interaction are central. The four main components of NPT interact with each other and the wider context of the intervention: coherence; cognitive participation; collective action; and reflexive monitoring [28, 43]. We combined the PBA and NPT [28] in a collaborative process (Figure 2) with an underpinning pragmatic epistemology, as spiritual health could be a topic particularly prone to ideological stalemates or conceptual impasse.

### 2.7 Reflexivity

As researchers, we recognise that our disciplinary backgrounds in public health and design, and our prior advocacy for co‐design, influenced how we approached and interpreted the co‐design process. O.W. and B.H. are practising GPs, with clinical and research experience in the topic, wider primary care and patient and public participation in research. A.O.D. is an expert in the development and evaluation of health and care interventions. M.A. is a social prescriber, with co‐design experience. M.P. and E.W. are working foundation doctors. O.W., M.P., P.M. and E.W. were relatively new to co‐design, while B.H. and A.O. are experienced within this field. We used strategies such as rotating facilitation roles, reflexive field notes and debrief meetings to examine how our assumptions, time constraints and institutional priorities affected decision‐making. We acknowledge that while our positions inevitably shaped the co‐design interactions and analytic choices, use of reflexive practices to triangulate findings enhanced the transparency and trustworthiness of this process evaluation.

### 2.8 Public and Patient Involvement (PPI)

PPI work before this project involved members of Voice Global [45], described above. Participants expressed the importance of person‐centred care, and the place of spiritual health within that. However, they had concerns about placing any additional burdens on NHS staff and systems and felt that doctors should not be expected to provide spiritual health support. A follow‐up consultation with VOICE members took place on 11th September 2025 to discuss the SHARP intervention and how best to disseminate and explain the SHARP project to patients and the public. A cartoon explaining SHARP training for use on GP practice screens is being developed.

### 2.9 Ethics Approval and Consent to Participate

Ethical approval was obtained from Newcastle University on 21st August 2024, and HRA approval was obtained on 25th October 2024, IRAS number 347636.

This paper addresses the following research question: How did the use of co‐design within the sensitive context of spiritual health shape outcomes at individual, group, process, and system levels, and what lessons does this offer for future co‐design work in value‐laden topic areas?

## 3 Results

The five workshops were attended by 38 participants in total, with 27 of these unique participants whose roles are listed in Table 3:

**Table 3:** Workshop participants’, roles.

Participants 1–6	General practitioner
Participants 7–8	Primary Care Clinician
Participants 9–10	Social prescribing worker
Participants 11–15	Spiritual health worker
Participants 16–18	Community Faith Based Organisation staff member
Participants 19–22	Member of the public or NHS patient
Participants 23–27	Any other interested person

There were 24 responses to post‐workshop evaluation forms, comments gave positive feedback that they felt the sessions were interesting and important, and that while they recognised everyone's time pressures, they could have spent more time on the discussions. Quantitative survey data are presented in Table 4.

**Table 4:** Results of post‐workshop evaluations.

Survey responses (*n* = 24)	Agree	Not sure	Disagree
My views and experiences were listened to	23 (95.8%)	1 (4.2%)	
My ideas and experiences were valued	23 (95.8%)	1 (4.2%)	
The workshop was interesting and engaging	24 (100%)		
The information was presented in a way that was easy to understand	24 (100%)		
The tasks were clear and manageable	20 (83.3%)	4 (16.7%)	
The workshop was relevant to my experiences	24 (100%)		
I feel the intervention we are developing is likely to be helpful for people like me	19 (79.2%)	5 (20.8%)	
The workshop was too short	18 (75.0%)	3 (12.5%)	3 (12.5%)
The workshop was too long	1 (4.2%)	3 (12.5%)	20 (83.3%)
Communication before the workshop was poor	1 (4.2%)	1 (4.2%)	22 (91.7%)
I found the workshop easily	20 (83.3%)	2 (8.3%)	1 (4.2%)^a^
Overall, I enjoyed the workshop	24 (100%)		

^a^ Not answered 1 (4.2%).

Of the 10 participants who attended more than one workshop, nine attended two workshops and one attended three workshops. A total of 24 post‐workshop evaluations were completed; 18 of these were completed by unique participants. Responses were three males (12.5%), 21 females (87.5%); one Buddhist (5.6%), seven Christians (38.9%), one Hindu (5.6%), three Humanists (16.7%), one Sikh (5.6%) and five with no religion (27.8%).

### 3.1 Findings

#### 3.1.1 People‐Level Outcomes and Processes

Participants described the workshops as providing a safe and rare opportunity to articulate their experience, values and concerns about spiritual health in ways that are not routinely voiced in their professional contexts.

It is so refreshing to be involved in conversations re spiritual care in Primary Care

(Participant 16, WS1)

However, participants appeared to be concerned about how others would respond to the topic, including the rest of the research team, and patients:

we don't normally go down this avenue, this is normally taboo.

(Participant 21, WS4)

Participants also shared worries about raising patients' expectations of receiving support around spiritual health without there being adequate resources:

*but there's nowhere I can actually refer to directly for general spirituality kind of issues.*.

(Participant 6, WS5)

For many, this opportunity to speak and be listened to functioned as a prerequisite for meaningful involvement, rather than an incidental benefit of participation. Participants appeared to dwell on aspects of spiritual health that appeared relevant to their own sense of identity, which suggested anxiety over not having been heard. Having a pre‐existing agenda and things that they *needed* to say appeared to come at the cost of what participants *could* contribute to the co‐design—they were unable to fully commit cognitively to the planned tasks before feeling heard on their pre‐existing agenda.

A small number appeared to express frustration when their personal viewpoints were not given extended attention, highlighting a common co‐design challenge in maintaining task focus while accommodating strongly held individual perspectives. In one 30‐min subgroup exercise, participants 25 and 8 spent a third of the time discussing their own beliefs about spiritual health before the facilitator redirected them to the task. Even with the use of patient stories and ‘think‐aloud’ prompts, facilitators often needed to steer discussion back from philosophical debate to practical design questions. Some participants needed space to express themselves, and therefore offering one‐to‐one meetings with a researcher allowed two participants the space they required to be heard and contribute. However, for one participant, the need to feel heard was a need to be heard by the group, rather than the researchers alone.

In the later workshops, participants appeared more excited about the developing training. Feeling recognised increased willingness to engage with structured activities, and the intentionally diverse mix of participants drew attention toward collaborative tasks and led to the emergence of some early ‘norm brokers’—a term we used to describe those who could identify norms within primary care, and how these could be mediated and developed via training to translate norms across settings.

#### 3.1.2 Group Process‐Level Outcomes: Participation, Trust, Hierarchy

At a group process level, Peter et al. [39] conceptualise evaluation as focusing on how the co‐design group works together, aligning with the SHARP project's aim of fostering equitable collaboration. Observers highlighted the lead facilitator's ‘soft skills’, including sensitivity to group dynamics and a relational speaking style, as contributing to an atmosphere of mutual respect.

Despite explicit attempts within the study design to tackle professional and social hierarchies, and promote equity of voice, including ground rules (Appendix 2), and equal thank‐you gifts, hierarchies appeared pervasive. GPs tended to cluster together, assumed leadership roles and their body language and tone signalled their status. Some participants signalled their professional or moral authority through references to long experience, additional roles or religious practice. Public contributors and those outside primary care reported feeling less confident and sometimes identified themselves as ‘outsiders’. The hierarchies observed reflect the existing hierarchies with wider society, and within primary care, with some deferring to clinicians' views:

I think that your opinion on this is much more beneficial than mine, as you work in the circles.

(Participant 26, WS3)

Where participants were mixed in terms of backgrounds in small groups, they were observed engaging collaboratively, building on one another's ideas and drawing on shared experiences of NHS practice, with moments of openness and respectful exchange of opinions. Small group work, consciously mixed, in face‐to‐face settings appeared to be most beneficial for achieving some equity of voice for the co‐design.

Group‐level psychological safety appeared variable: some discussions were energised by disagreement, while in other cases participants hesitated to ask basic questions until prompted. The short duration of workshops may have limited the deeper trust‐building required for sustained psychological safety. For some participants, however, the workshops did foster a sense of safety and inclusion:

I really felt safe and welcome and that my voice was heard. There are many places where that is not how I am left feeling so I really appreciate your thoughtfulness and care.

(Participant 24, WS1)

In practice, achieving full equity of voice within such a diverse group was challenging, and limited by preconceived and established hierarchies.

#### 3.1.3 Research Process‐Level Outcomes: The Push/Pull of Pragmatism

Those who attended the workshops had all disclosed an interest in the topic and may therefore have preferred longer meetings; indeed, 75% of those completing the evaluation survey felt the workshops were too short. However, the 1‐h lunchtime format enabled working primary care staff and those with caring responsibilities to attend, providing access to ‘coal face’ voices.

it felt rushed however I appreciate that time is limited for some.

(Participant 9, WS5)

To obtain a diversity of perspectives and minimise repeated commitments, each workshop involved a different mix of participants, although a ‘spine’ of continuity meant that each workshop had some members of the previous ones, to attempt to bring some continuity and shortcut to group formation. While this broadened input, the development of group identity, we observed cohesion and continuity appeared reduced as a result. Some participants disclosed feeling less confident contributing, perceiving others as more knowledgeable or better positioned to participate, which at times constrained their engagement and highlighted the need for greater continuity or more explicit efforts to bring participants up to date.

Bring people up to date with information gathered from previous workshops and begin to explain the next steps.

(Participant 15, WS5)

Researchers and workshop attendees alike recognised the pros and cons of having a different mix of participants in each workshop. Increasing the number and variety of contributions may have bolstered the intervention's relevance across primary care settings; however, this may have come at the cost of deeper understanding of the aims of the intervention:

I think it may be beneficial to offer the four parts of the training workshop to the same set of people, so that their input comes from having greater insight into the overall picture.

(Participant 26, WS3)

Workshop settings also shaped the co‐design process and generated stronger engagement. Observers noted the formation of *‘social bonds’* and a more *‘natural flow to conversations’* (Observer 1, WS1) during in‐person meetings, whereas online meetings tended to produce *‘less of a feeling of discussion and more individual voicings of thoughts’* (Observer 3, WS2). At the same time, online workshops appeared to reduce professional clustering, which was particularly marked in the first in‐person session held in a formal, traditional room where professionals clustered in their groups, and members of the public sat further away from researchers. Observers suggested that this spatial arrangement, as well as formal academic atmosphere, contributed to a power dynamic in which clinical voices dominated despite ground rules encouraging equality.

As discussed above, the need of some participants to feel heard on their own terms had practical consequences for the co‐design process, at times limiting progress on structured tasks.

Despite the greater ease of attendance, online workshops were less well attended than in‐person sessions, possibly reflecting higher commitment among those attending face‐to‐face, the more concrete feel of in‐person engagement, more difficulty establishing the safety and rapport needed for value‐laden topics, or concerns about confidentiality when discussing a sensitive topic online.

#### 3.1.4 System and Sustainment‐Level Outcomes: Embedding SHARP Into Practice

Throughout the co‐design process, participants identified barriers to sustaining change alongside the practical conditions they felt would support integration of spiritual health training into primary care. We observed a solutions‐based and positive approach to identifying barriers to implementation of the training. Spiritual health was relevant to the participants, but they recognised that change would be challenging to implement in practice. Across the workshops, participants adopted a largely solution‐focused stance, emphasising the importance of recognising appropriate opportunities for these conversations, allowing practice staff to take ownership, ensuring protected time and embedding activities within existing practice structures.

Participants' contributions reflected an awareness of how different parts of the system might work together in practice, which added depth to the co‐design of the training.

So, you know, we certainly can have half an hour, or 45 min, together as a practice, at least certain groups of the practice, pharmacists, or GPs, or whatever. So yeah, there is opportunity, sometimes, to do this sort of thing.

(Participant 6, WS5)

This breadth of perspective helped ensure that the resulting training materials were relevant to whole‐team delivery and sensitive to the realities of multidisciplinary primary care settings.

Seeing clear benefits for patients was viewed as a key motivator for clinician engagement with the co‐design tasks, with several participants noting that open conversations could enhance rapport, efficiency, and care quality.

You know, if we're [primary care] convinced that something is worth doing from the patient's perspective, we're more likely to not be afraid to have a go at it.

(Participant 27, WS5)

## 4 Discussion

### 4.1 Summary of Findings

We used the framework proposed by Peters et al.'s [39], to reflect on the co‐design process and to identify learning relevant to the use of co‐design in spiritual health. This analysis highlights how the process generated outcomes at individual, process, and system levels, while also revealing key strengths and challenges, particularly in relation to hierarchy, shared language and identity tensions when working with sensitive topics. These reflections draw on our experience of developing a spiritual health training for primary care and are intended to inform future intervention development using co‐design, both within spiritual health and in other sensitive areas.

The co‐design process highlighted a strong need for participants to express views and feel heard before they were able to engage with structured activities. Providing space for this expression was necessary to establish safety, trust and psychological comfort in a topic where language and attachment to preferred practices or ideologies presented anticipated challenges. Persona‐based discussions and narrative techniques enabled participants to articulate views indirectly, reducing personal vulnerability and supporting flexible, structured conversations that validated diverse beliefs and experiences.

The use of short meetings and a combination of online and in‐person workshops increased accessibility and enabled participation from a wider range of professional and public contributors, generating a breadth of perspectives. However, reliance on a largely non‐static group limited the development of stability and psychological safety and increased the need for participants to re‐establish their positions and attachments to preferred areas of discussion. Greater continuity of participants, allowing progression through Tuckman's stages of forming, storming, norming and performing [46], may have generated deeper co‐design data, but this would likely have come at the cost of reduced breadth of input.

Longer meetings may have further supported participants' sense of being heard but would have risked excluding those with significant time pressures. One‐to‐one meetings with the research team supported some participants to move beyond strongly held professional attachments, although this was not effective for all, and may represent a useful supplementary strategy when co‐designing in sensitive topic areas.

The use of a mixed co‐design group, including professionals, members of the public, carers, social prescribers, GPs, faith‐based workers, chaplains and those with managerial roles, strengthened the system‐level insights generated through the process, and provided ecological validity to the training. Bringing together participants with different roles and perspectives enabled consideration of how responsibility for spiritual health could be shared across the wider primary care team and embedded within existing systems and pressures. This supported discussion of sustainability as a collective endeavour and informed the development of training content oriented towards whole‐team implementation in real‐world practice.

While the diversity of participants was a key strength of the co‐design process, pluralism also presented challenges, requiring careful navigation of identity, culture, professional boundaries and hierarchy through facilitation and guiding principles. Running separate workshops for clinical and non‐clinical participants may have reduced some concerns about hierarchy and equity, as mixed groups can create inhibition in both directions. In our workshops, non‐clinical participants sometimes deferred to GPs, while clinicians may also have moderated their contributions in the presence of a non‐peer audience.

However, although the primary users of the training are primary care clinicians, consultations are relational encounters that require attention to the patient voice as an equal partner, a principle recognised in spiritual health training within palliative care. By using mixed groups, the co‐design process reflected the realities of practice, where spiritual health is co‐experienced, and provided ecological validity. While enabling equal voice remained challenging, the presence of established hierarchies mirrored real‐world power distributions and generated additional insight relevant to training design.

Through the workshops, we saw the emergence of what we termed ‘*norm brokers’*—participants who can champion and normalise the topic in practice. Participants took a solution‐focused orientation, which was likely shaped by the co‐design approach itself, as participants were recruited for interest in the topic and willingness to engage. Such motivated participants were necessary to support productive co‐design work and the development of feasible outputs. However, this also meant that the process may have been less well positioned to uncover additional barriers to change beyond those already identified through the wider background and evidential research, which might have emerged had the co‐design been structured or sampled differently.

Through use of a flexible, user focussed, supported approach (PBA), underpinned by evidence and theory (NPT), a co‐designed training has been developed, peer reviewed and is ready for pilot and evaluation.

### 4.2 Strengths and Limitations

Integrating the PBA with a robust evidence base and pedagogical and behaviour‐change theories enabled the development of a pragmatic, accessible training intervention tailored to real‐world primary care practice. Participant engagement was high, with many attending more than one workshop despite significant time pressures.

In designing the co‐design process, we prioritised breadth of input over depth, using a combination of online and in‐person workshops to maximise participation. This limited the depth of data generated within individual sessions, as each workshop required group formation and reiteration of shared ground rules, and may have contributed to recurring early‐stage group dynamics, such as revisiting preferred practices. Partial continuity of participants across workshops provided some stability and supported progression towards norming and performing [46], while still allowing new perspectives to be introduced.

All participants expressed an interest in spiritual health, which may bias the co‐design towards those already receptive to training. Using Appleby et al.'s framework, [14, 47] which identified four types of attitudes towards spiritual health in primary care from GPs, this suggests greater representation of ‘embracing’ participants, with fewer voices from those who may be more ‘pragmatic’ or ‘guarded’ and potentially stand to benefit most from training. Participants who were wholly rejecting of spiritual health were not included, as repeated discussion of the topic's relevance within primary care risked undermining the feasibility of co‐design. While attempts were made to recruit a range of perspectives, this was not formally assessed and may limit transferability. At the same time, recruiting motivated participants enabled the co‐design process to proceed without becoming stalled in philosophical debate.

We were limited that the post‐workshop evaluation forms were optional, meaning some participants may have had negative feelings about the co‐design workshops, such as feeling unheard, frustrated or strained that they chose not to share.

### 4.3 Comparison With Other Literature

SHARP is not the first time that co‐design has been used in the area of spiritual health. The DIGNISPACE intervention [48], and The Examen Tu Salud [49] used co‐design with young people on the topic; however, there was no formal evaluation of how the co‐design was experienced by those involved, nor whether that team faced similar challenges to SHARP [48, 49].

Issues with hierarchy and power are not unique to spiritual health and are recognised as a barrier to collaboration within co‐design in mental health [50], research around adoption and children in care [51, 52, 53, 54], and research into gender and sexual minorities [55], neurodivergence, or where these overlap [56]. Spiritual health may not have the historical inequalities of mental health services, but issues at the intersections of inequality or minority status [50, 55] could affect contributions and safety within co‐design groups. This can lead to the exclusion of stakeholders in research, with policies therefore developed without their input [52]; however, while there needs to be additional care, stakeholder involvement is vital [56]. Most participants brought a variety of identities to the SHARP workshops, often having complex careers leading to multiple professional, patient and caring roles, and different experiences within research. These will all have affected participants' engagement with the co‐design, and how power and hierarchy worked within the groups. Therefore, while there have been other uses of co‐design in sensitive topics, there have not been any published evaluations sharing learning from the use of co‐design in value‐laden topic areas. By publishing our reflections from SHARP, hopefully the challenges of the co‐design process could be understood and improved in the future, not just for spiritual health, but for other value‐laden topics too.

### 4.4 Recommendations

Navigating hierarchy and power sharing within co‐design is complex [50]. This is the first paper we are aware of that reflects on that navigation within a topic of sensitivity, strong views and linked to identity, like spiritual health. For future use of co‐design in spiritual health, we recommend greater reflection and cognisant navigation of power and intersectionality of identities within the workshop group participants. This greater reflection, evaluation and discussion around the complexity of research participation and co‐design in spiritual health may not only benefit that topic, but also other topics where culture, identity, power, trauma, minority status and strong opinions intersect, such as gender research, sexuality, neurodivergence or adoption and looked‐after children or former looked‐after children. Greater understanding of how to validate and listen to the *need* to share strong opinions early, giving early priority to relational investments, may allow participants to meet their expressive needs before group work. This may enhance collaboration between the participant, the group, the project and the research team, and lead to better co‐design. This paper reports a process evaluation of co‐design in a sensitive, or taboo, topic area. The findings are primarily intended to inform research practice: specifically, how researchers can structure co‐design to support equitable participation, manage power dynamics, and navigate identity and values in contested or taboo subject areas. Clinical implications of the SHARP training intervention itself are intended to be reported in subsequent papers reporting the pilot and evaluation.

## 5 Conclusion

This study examined the use of co‐design to develop a training intervention within a sensitive and value‐laden topic area. Using the PBA [22], supported by theory and evidence, provided a flexible yet structured way to attend to user journeys, support dialogue and progress towards a feasible training output. Creating safe, equality‐focused spaces where participants could express views and feel heard was central to enabling engagement in this context.

The co‐design process also highlighted challenges inherent in collaborative work on sensitive topics. Participants' need to articulate strongly held views and feel listened to functioned as a prerequisite for engagement with structured design activities, rather than a disruption to them. Hierarchies, professional identities and practical constraints shaped participation and required active facilitation. Decisions around group composition, continuity, workshop length and the use of supplementary one‐to‐one discussions involved trade‐offs between breadth of input, depth of engagement and feasibility, which must be considered in advance when designing similar work.

Overall, this study contributes methodological insight into how co‐design can be structured to support equitable participation, manage power and expressive needs and enable system‐level thinking in sensitive contexts. These reflections are intended to inform future co‐design efforts in spiritual health and other areas where identity, values and power intersect.

## Author Contributions

**Ishbel Orla Whitehead:** conceptualisation, investigation, writing – original draft, funding acquisition, methodology, validation, visualisation, writing – review and editing, formal analysis, project administration, data curation, supervision, resources. **Mark Adley:** writing – original draft, writing – review and editing, formal analysis, project administration, data curation, investigation, visualisation. **Alexandra Thompson:** formal analysis, project administration, investigation. **Elizabeth Westhead:** writing – review and editing, formal analysis, project administration, investigation. **Marina Politis:** writing – review and editing, formal analysis, project administration, data curation. **Philip Mordue:** formal analysis, data curation, project administration. **Amy O'Donnell:** funding acquisition, conceptualisation, methodology, writing – review and editing, supervision, validation. **Barbara Hanratty:** conceptualisation, investigation, formal analysis, writing – review and editing, validation, supervision, methodology, funding acquisition.

## Ethics Statement

Ethical approval was sought and obtained from Newcastle University on 21st August 2024, IRAS number: 347636.

## Consent

Participants were asked to give their informed consent for participation, analysis and publication before participating.

## Conflicts of Interest

The authors declare no conflicts of interest.

## Supporting information

Supporting File 1

Supporting File 2

Supporting File 3

Supporting File 4

Supporting File 5

Supporting File 6

Supporting File 7

## Data Availability

Data are saved on Newcastle University secure servers and may be available in negotiation with the first author. While participants were not consented to allow public sharing of this data, data are available upon reasonable request to the authors.
