# Supplementary material for: “We Don't Normally Go Down This Avenue; This Is Normally Taboo”: Using Co‐Design to Develop a Training Intervention for Spiritual Health in Primary Care
Source: Health Expect. 2026 Jun 21;29(3):e70737. doi: 10.1111/hex.70737 (PMC13283352; doi:10.1111/hex.70737)
Supplement: Supplementary file 1 — Supporting File 1 [file HEX-29-e70737-s003.docx]

# SHARP Workshop 1 Agenda

**12:30-12:40 | Welcome & Introductions**

- Quick round of names and roles
- Review of the ground rules (document 2)

**12:40-12:45 | Overview of the SHARP intervention training**

- Introduction of the guiding principles (document 3)
- Discussion of guiding principles

*The SHARP training intervention is training for the primary care team (those who work in GP practices) to discuss spiritual health with patients, and then make suggestions of how the patients could look at meeting those needs.*

**12:45-1:15 | User Journey Exercise- Mina**

Small group/pairs discussion of the persona (document 4)

**1:15-1:25 | Reflection**

**1:25-1:30 | Next Steps**

Post workshop survey

# SHARP workshop 2 Agenda

**11:00-11:10 | Welcome & Introductions**

Quick round of names and roles

Review of the ground rules and guiding principles (documents 2 and 3)

**11:10-11:20 | Overview of the SHARP intervention training**

**11:20-11:25 | How to present the HOPE tool**

**11:25-11:45 | Patient stories ‘think aloud’**

**11:45-12:00 | Plenary**

Next Steps

Post workshop survey

# SHARP Workshop 3 Agenda

**12:30-12:40 | Welcome & Introductions**

- Quick round of names and roles
- Review of the ground rules (document 2)

**12:40-12:45 | Overview of the SHARP intervention training**

- Review of the guiding principles (document 3)
- Review of the aims and objectives of the training (document 4)

*The SHARP training intervention is training for the primary care team (those who work in GP practices) to discuss spiritual health with patients, and then make suggestions of how the patients could look at meeting those needs.*

**12:45-1:15 | Review of the prototype**

Small group/pairs discussion of the paper prototype

**1:15-1:25 | Plenary**

**1:25-1:30 | Next Steps**

Post workshop survey

# SHARP Workshop 4 Agenda

**12:30-12:40 | Welcome & Introductions**

- Quick round of names and roles
- Review of the ground rules (document 2)

**12:40-12:45 | Overview of the SHARP intervention training**

- Review of the guiding principles (document 3)
- Review of the aims and objectives of the training (document 4)

*The SHARP training intervention is training for the primary care team (those who work in GP practices) to discuss spiritual health with patients, and then make suggestions of how the patients could look at meeting those needs.*

**12:45-1:15 | Review of the prototype**

Small group/pairs discussion of the prototype re presentation of the HOPE tool

**1:15-1:25 | Plenary**

**1:25-1:30 | Next Steps**

Post workshop survey

# SHARP Workshop 5 Agenda

**12:30-12:40 | Welcome & Introductions**

- Quick round of names and roles
- Review of the ground rules (document 2)

**12:40-12:45 | Overview of the SHARP intervention training**

- Review of the guiding principles (document 3)
- Review of the aims and objectives of the training (document 4)

*The SHARP training intervention is training for the primary care team (those who work in GP practices) to discuss spiritual health with patients, and then make suggestions of how the patients could look at meeting those needs.*

**12:45-1:10 | Review of the prototype**

Review of the prototype

Small group/pairs discussion of the prototype re presentation of the HOPE tool

Pairs using the questions

Creative activity for presentation

**1:10-1:25 | Plenary, including any suggestions for dissemination and evaluation?**

**1:25-1:30 | Next Steps**

Post workshop survey
