# Supplementary material for: “We Don't Normally Go Down This Avenue; This Is Normally Taboo”: Using Co‐Design to Develop a Training Intervention for Spiritual Health in Primary Care
Source: Health Expect. 2026 Jun 21;29(3):e70737. doi: 10.1111/hex.70737 (PMC13283352; doi:10.1111/hex.70737)
Supplement: Supplementary file 2 — Supporting File 2 [file HEX-29-e70737-s004.docx]

# Guiding Principles

# The SHARP Training will be:

- Using a working definition of spiritual health:

Spiritual health is a broad concept, as diverse as people themselves. We use a working definition of spiritual health developed by UK General Practitioners and social prescribers:

- self-actualisation, peace, purpose and meaning;
- transcendence, connectivity and relationships beyond the self;
- expressions of spirituality (including practice)
- Based on the evidence that spiritual health is an important part of whole person health
- Based on the evidence of training needs for the primary care team around the topic of spiritual health
- Practically useful
- Patient focused
- Understanding of diversity
- Encouraging self reflection for both practitioner, and patient
- Using the HOPE tool as a tool box, and is not prescriptive
- Giving an overview of religions or spiritual paths encountered in UK primary care

# Ground Rules

- This is a diverse group of people who are all equally valuable. Every voice matters. Everyone here brings valuable lived experience or insight.
- We may all have different understandings regarding spiritual health. We will all have different experiences of spiritual health, and these different perspectives are valued and respected here.
- We are here to find a path forwards and not get stuck in any one debate.
- We will be respectful, considerate of others and polite at all times. However, we are empowered to be clear if something is not working for us, or if we need to take a break, or disagree.
- We’re here to learn what works and what doesn’t—be as honest and real as you can*.*
- The design of the SHARP training is not fixed—we’re shaping it together. However, we do have a lot of evidence about what should be included, and the training will be ‘evidence based’.
- We’ve got a plan (agenda), but the best insights often come from unplanned moments. We will endeavour to balance the need to make our way through topics in a timely manner with exploration of interesting and useful tangents.
- Your thoughts help us make this training better for real people.

Spiritual Health Awareness and Recommendations in Primary care (SHARP) training
Aims and Objectives

# Aims

1. To upskill the primary care team around the topic of spiritual health, and inspire teams to consider how spiritual health needs can be identified, and make recommendations to patients.
2. To address the barriers to inclusion of spiritual health within routine primary care
3. To prompt reflection about spiritual health in patient care
4. To prompt reflection about spiritual health in practitioner wellbeing

# Objectives

## Knowledge-Based Objectives

1. To introduce spiritual health, what it is, and why it matters to health
2. Recognise common spiritual needs, or aspects of spiritual distress
3. To introduce the HOPE tool as a ‘toolbox’ of useful questions

## Skill-Based Objectives

1. Consider how spiritual health could be integrated within practice
2. Identify spiritual health needs within patient stories
3. Consider communication techniques that facilitate respectful and compassionate spiritual conversations.
4. Understand that patients may wish to access chaplains, faith leaders, or other spiritual care providers, and consider how this could happen within your own team

## Attitude-Based Objectives

1. Reflect on personal beliefs and biases and how they influence patient care.
2. Foster cultural humility and respect for diverse spiritual and religious worldviews.
3. Develop empathy and sensitivity to spiritual distress or existential suffering in patients.

## Systems Objectives

1. Understand ethical and legal boundaries related to spiritual discussions in healthcare.
2. Explore strategies to create a spiritually supportive environment within your own team
3. Promote interdisciplinary collaboration in addressing spiritual health.
