# Supplementary material for: “We Don't Normally Go Down This Avenue; This Is Normally Taboo”: Using Co‐Design to Develop a Training Intervention for Spiritual Health in Primary Care
Source: Health Expect. 2026 Jun 21;29(3):e70737. doi: 10.1111/hex.70737 (PMC13283352; doi:10.1111/hex.70737)
Supplement: Supplementary file 3 — Supporting File 3 [file HEX-29-e70737-s007.pdf]

Are you interested in spiritual health?

If you live in the UK, are over 18, and want to help with a research project about spiritual health – we would love to hear from you

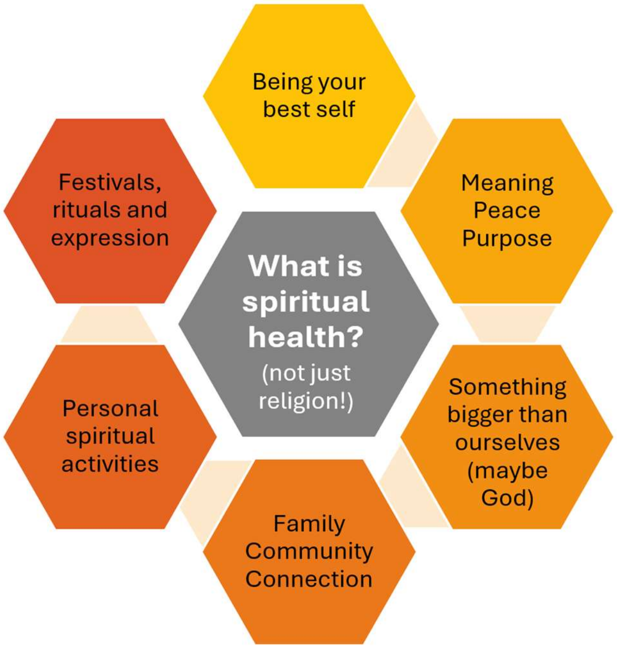

We are a team from Newcastle University studying how healthcare services can work with patient's feelings and beliefs to promote health.

We need interested people (patients, carers, social prescribers, chaplains, parish nurses, experts by experience, spiritual organisation members, the public, and primary care clinicians) to help us develop training in this area for health professionals.

The meetings will be either face to face, or on the computer, depending on which suits you, and there will be **four to six** of them. Each one lasts **one hour**. You could attend one, some, or all, of these meetings

To register your interest, visit bit.ly/sharp-workshops

Please contact the study team for further information:
mark.adley@newcastle.ac.uk

JOHN TEMPLETON FOUNDATION
Inspiring Awe & Wonder

Newcastle University
