# Supplementary material for: “We Don't Normally Go Down This Avenue; This Is Normally Taboo”: Using Co‐Design to Develop a Training Intervention for Spiritual Health in Primary Care
Source: Health Expect. 2026 Jun 21;29(3):e70737. doi: 10.1111/hex.70737 (PMC13283352; doi:10.1111/hex.70737)
Supplement: Supplementary file 4 — Supporting File 4 [file HEX-29-e70737-s001.docx]

**Recruitment and Sampling: supplemental information**

Participants were purposely recruited from 22^nd^ February 2025 to 22^nd^ March 2025. Email invitations were sent out throughout the UK to care boards, practices, health boards, clinical research networks, primary care professional networks, social prescribing organisations, and nationwide organisations representing faith groups, such as the Muslim Council, Jewish care, Humanists UK, etc., as well as Voice UK (https://voice-global.org/). Invitations specifically asked for participation from people with an interest in spiritual health (see Appendix 3 for recruitment flyer), with eligibility criteria below.

| **Inclusion criteria** | **Exclusion criteria** |
| --- | --- |
| *People with an interest in the topic of spiritual health in primary care*  *(For in-person workshops) able to travel to Newcastle-upon-Tyne* | *People who did not disclose an interest in spiritual health.*  *Individuals who were assessed as unable, or not willing, to understand or follow the workshop ground rules and guiding principles.*  *Those who were assessed as high risk of fraudulent participation, e.g. to be outside the UK, and not have a genuine interest in spiritual health in primary care in the UK.* |

This approach to recruitment was to ensure a range of voices in terms of professions, religious and cultural background, but also to support psychological safety within workshops and focus discussion on the training content and minimise debate around issues that we had pragmatically decided to move on from, for example definitions of spiritual health, whether spiritual health is part of health at all, or which of the range of tools for spiritual discussion was best. These decisions were taken pragmatically in line with the latest scientific evidence, so that the discussion could be dynamic and focussed on forward movement for the training. Those interested in taking part were directed to an online sign-up form, which collected demographic information such as job role, geographical location, and whether they would be able to attend face-to-face workshops, as well as some indication as to their interest in the topic. Invitations were then sent out for each workshop from this list of potential participants, with selection discussed in the whole research team. Participants were offered a £50 voucher for each workshop attendance was offered in gratitude, and steps were taken to identify fraudulent study participants (see below). All members were given the same gift, to reinforce equality of contribution to the co-design, regardless of role outside the project.

**Identifying fraudulent research participants**

Any doubts to be discussed as a team at the earliest opportunity

namenumber@gmail addresses will not be invited.

Other non-work, or non-organisational email addresses will be invited to a screening discussion with a member of the tea, The authenticity/inauthenticity of their response to be discussed as a team. This can be followed by further questions to ascertain their knowledge of the topics of UK primary care and spiritual health via phone or email.

Non-UK participants will be barred.

Videos must be on.

The voucher will be given unconditionally to genuine participants, even if they withdraw. However, it will be made clear that if we believe that they are not a genuine participant, then the interview will be terminated without remuneration. All efforts will be made through questioning to clarify this early.

Check early on that there is a genuine understanding of primary care, the NHS, and how they fit within that.
