# Supplementary material for: “We Don't Normally Go Down This Avenue; This Is Normally Taboo”: Using Co‐Design to Develop a Training Intervention for Spiritual Health in Primary Care
Source: Health Expect. 2026 Jun 21;29(3):e70737. doi: 10.1111/hex.70737 (PMC13283352; doi:10.1111/hex.70737)
Supplement: Supplementary file 5 — Supporting File 5 [file HEX-29-e70737-s006.docx]

|  |  | Notes |
| --- | --- | --- |
| Participation Patterns | Who speaks to whom, frequency of interactions, nature of exchanges |  |
| Body Language | Posture, facial expressions, physical positioning in space |  |
| Energy levels | Engagement patterns, enthusiasm, attention spans, energy shifts |  |
| Power Dynamics | Who influences decisions and directs activities |  |
| Inclusion | Whether all voices are heard and valued equitably |  |
| Psychological Safety | Comfort in sharing ideas without fear of criticism |  |
| Conflict Resolution | How disagreements are addressed and resolved |  |
| Collaborative Synergy | Evidence of building on each other's contributions |  |

|  |
| --- |

Jobs:

- Sketch out who sits where please
