# Supplementary material for: “We Don't Normally Go Down This Avenue; This Is Normally Taboo”: Using Co‐Design to Develop a Training Intervention for Spiritual Health in Primary Care
Source: Health Expect. 2026 Jun 21;29(3):e70737. doi: 10.1111/hex.70737 (PMC13283352; doi:10.1111/hex.70737)
Supplement: Supplementary file 6 — Supporting File 6 [file HEX-29-e70737-s005.pdf]

| | SURVEY QUESTION | PULL-DOWN OPTIONS |
|---|---|---|
| 1 | What is your name? | |
| 2 | Would you describe your role as: | General Practitioner / Social prescribing worker / Primary Care Clinician / Member of the public or NHS patient / Community Faith based organisation staff member / Community Faith based organisation volunteer / Spiritual health worker / Any other interested person (please detail below) |
| 3 | Any other interested person: | |
| 4 | Are you a: | Woman / Man / Self describe (please specify) |
| 4 | Self describe (please specify) | |
| 5 | Would you describe yourself as being part of one of these groups? | No religion / Christian / Humanist / Buddhist / Sikh / Hindu / Muslim / Jewish / Other (please specify) |
| 5 | Would you describe yourself as being part of one of these groups? - Other (please specify) | |
| 6 | What are the most important things we need to change in the SHARP training? | |
| 7 | How did you feel about today's workshop? | |
| 8 | Do you agree or disagree with these statements? | |
| 8.1 | My views and experiences were listened to | Agree / Not Sure / Disagree |
| 8.2 | My ideas and experiences were valued | Agree / Not Sure / Disagree |
| 8.3 | The workshop was interesting and engaging | Agree / Not Sure / Disagree |
| 8.4 | The information presented in a way that was easy to understand | Agree / Not Sure / Disagree |
| 8.5 | The tasks were clear and manageable | Agree / Not Sure / Disagree |
| 8.6 | The workshop was relevant to my experiences | Agree / Not Sure / Disagree |
| 8.7 | I feel the intervention we are developing is likely to be helpful for people like me | Agree / Not Sure / Disagree |
| 8.8 | The workshop was too short | Agree / Not Sure / Disagree |
| 8.9 | The workshop was too long | Agree / Not Sure / Disagree |
| 8.10 | Communication before the workshop was poor | Agree / Not Sure / Disagree |
| 8.11 | I found the workshop easily | Agree / Not Sure / Disagree |
| 8.12 | Overall, I enjoyed the workshop | Agree / Not Sure / Disagree |
| 9 | What parts of the workshop did you enjoy the most? | |
| 10 | Was there anything you found confusing or frustrating? | |
| 11 | What could we do to improve the next workshop? | |
| 12 | Is there anything you would have liked more time or support with? | |
| 13 | Please add any further thoughts on the workshop process, or the training | |
