# Supplementary material for: “We Don't Normally Go Down This Avenue; This Is Normally Taboo”: Using Co‐Design to Develop a Training Intervention for Spiritual Health in Primary Care
Source: Health Expect. 2026 Jun 21;29(3):e70737. doi: 10.1111/hex.70737 (PMC13283352; doi:10.1111/hex.70737)
Supplement: Supplementary file 7 — Supporting File 7 [file HEX-29-e70737-s002.docx]

**Template: Table of changes**

How to use this table of changes: As you collect views on each intervention element or feature, extract all negative and positive comments and log them in the appropriate columns. Positive comments won’t need to be acted on, but are useful to record to balance negative views.

For negative comments, suggest solutions in the ‘Possible Change’ column. Then use the ‘Reason for change’ column to record why this change should be made. You can use the coding framework below, create your own codes or write freely if none of the categories apply. Some changes can be made immediately (e.g. EAS or REP), whereas you might want to discuss others (e.g. IMP) in your team. You can use the codes as a way to speed up the process of making changes, as you won’t need to discuss every change.

Use the final column to prioritise which changes to make first (MoSCoW; Must have, Should have, Could have, Would have). These can also be discussed in your team if needed. An example excerpt from a completed table is given below the template table.

| Coding framework | | |
| --- | --- | --- |
| Code | **Stands for** | **Means** |
| IMP | Important for behaviour change | This is an important change that is likely to impact behaviour change or a precursor to behaviour change (e.g. acceptability, feasibility, persuasiveness, motivation, engagement), and/or is in line with the Logic Model, and/or is in line with the Guiding Principles For example, participants appear unconvinced by an aspect of the intervention, so you decide to add motivational examples. |
| EAS | Easy and uncontroversial | An easy and feasible change that doesn’t involve any major design changes. For example, a participant was unsure of a technical term, so you add a definition. |
| REP | Repeatedly | This was said repeatedly, by more than one participant. |
| EXP | Experience | This is supported by experience. Please specify what kind of experience, for example:   1. PPIs agree this would be an appropriate change. 2. Other stakeholders (e.g. practitioners, providers, topic specialists) agree that this would be an appropriate change. 3. Literature: This is supported by evidence in the literature. |
| NCON | Does not contradict | This does not contradict experience (e.g. evidence), or the Logic Model, or the Guiding Principles |
| NC | Not changed | It was decided not to make this change. Please explain why (e.g. it would not be feasible; or only one person said this). |

| ***SHARP training*** | | | | | | |
| --- | --- | --- | --- | --- | --- | --- |
| ***Intervention element*** | ***Negative Comments*** | ***Positive Comments*** | ***Possible Change*** | ***Reason for change*** | ***Agreed change / NC*** | ***MoScoW*** |
| **Patient Stories / Vignettes** | Contradictions (e.g., “housebound” but also active in groups) | Stories seen as rich, realistic, and discussion-provoking | Edit text for internal consistency | IMP, REP |  | Must |
|  | Limited diversity (e.g., all appear White, heteronormative) | Useful range of scenarios | Add further ethnic/cultural diversity- to be discussed | IMP, REP, EXP |  |  |
|  | AI images distracting (e.g., “angry Michael,” “dead-looking baby”) | Some liked neutral line drawings | Replace with more neutral drawings, photos, or short audio/video clips | REP, EAS |  |  |
|  | Discussion can drift into purely medical debates (e.g., acne treatment for David) | Scenarios prompt useful reflection | Add facilitation notes + “cue checklist” (loss of purpose, isolation, values) | IMP, REP |  |  |
|  | Lack of explicit focus on spiritual trauma | Good at showing loneliness/purpose issues | Revise cases | IMP, EXP |  |  |
| **HOPE tool** | Too long for 10–15 min GP consults | Starting with “Hope” is inclusive | Provide abridged “HOPE-Lite” (1 bolded Q per letter) + full version for chaplains/social prescribers |  |  |  |
|  | “O = Organised Religion” alienates non-religious | Validated, widely recognised tool | Reframe as “Organised Religion or Belief Systems”; add secular examples |  |  |  |
|  | Patients may shut down at “O” or “P” (“I’m not religious/spiritual”) | “Hope” and “Meaning” questions seen as powerful | Add training on pivoting to values/meaning/purpose if patient rejects “O”/“P” |  |  |  |
|  | Acronym can feel rigid or off-putting | Helpful memory aid for some | Encourage flexible language use; show real-world transcripts of “HOPE in action” |  |  |  |
| **Training definitions** | Too focused on religion; risks excluding secular patients | Participants value inclusivity | Define spirituality broadly (awe, nature, music, values, identity) with everyday examples |  |  |  |
| **Training content** | Too abstract/dry (essay-like) | Participants want engaging, short format | Use patient quotes, stories, and examples rather than long text |  |  |  |
|  | Unclear boundaries → fear of complaints (e.g., offering prayer) | Participants value coverage of legal/pro guidance | Add module on safe boundaries and regulator guidance |  |  |  |
|  | Limited self-reflection | Participants want to build humility and awareness | Include reflection exercise: “What gives *you* hope/meaning?” |  |  |  |
|  | Risk of ignoring cues in short consults | Recognition of cues seen as critical | Add “cue checklist” and role-play examples |  |  |  |
| **Presentation style** | Text-heavy, risk of disengagement | 30-minute length acceptable | Make training visual (slides with images, short video clips, audio narrations) | REP, EAS |  |  |
|  | Photos/cartoon debate (mixed views) | Some like neutrality of cartoons, others authenticity of photos | Offer flexible formats (cartoons, photos, or audio); avoid loaded/distracting imagery | REP |  |  |
|  | Role-play could become farcical | Role-play seen as powerful teaching | Provide structured “good vs poor” consultation videos with facilitation guide | IMP, REP |  |  |
| **Presentation delivery** | Lack of follow-up support | Participants value team-based reflection | Provide post-training tools: reflection sheets, handouts, lunchtime discussion prompts | IMP, REP |  |  |
|  | Tone risks trivialising (humour used poorly) | Sensitive topic respected when serious | Keep tone respectful; allow light humour only for relief | IMP, EXP |  |  |
|  |  |  |  |  |  |  |
|  |  |  |  |  |  |  |
|  |  |  |  |  |  |  |
|  |  |  |  |  |  |  |
